# Methyl 1-[(6-meth­oxy-5-methyl­pyrimidin-4-yl)meth­yl]-1*H*-benzo[*d*]imidazole-7-carboxyl­ate: a combined X-ray and DFT study

**DOI:** 10.1107/S2414314623000251

**Published:** 2023-01-12

**Authors:** Adrian Richter, Richard Goddard, Roy Schönefeld, Peter Imming, Rüdiger W. Seidel

**Affiliations:** a Institut für Pharmazie, Martin-Luther-Universität Halle-Wittenberg, Wolfgang-Langenbeck-Str. 4, 06120 Halle (Saale), Germany; b Max-Planck-Institut für Kohlenforschung, Kaiser-Wilhelm-Platz 1, 45470 Mülheim an der Ruhr, Germany; Goethe-Universität Frankfurt, Germany

**Keywords:** crystal structure, DFT calculations, benzimidazole, pyrimidine, tuberculosis

## Abstract

The title compound, C_16_H_16_N_4_O_3_, was obtained as a side product during the synthesis of the previously reported anti­tubercular agent *N*-(2-fluoro­eth­yl)-1-[(6-meth­oxy-5-methyl­pyrimidin-4-yl)meth­yl]-1*H*-benzo[*d*]imidazole-4-carboxamide and structurally characterized by X-ray crystallography and computational methods. In the crystal (space group *P*2_1_/*n*, *Z* = 4), the title compound adopts a twisted conformation with a dihedral angle between the benzimidazole and pyrimidine mean planes of 84.11 (3)°. The carboxyl­ate group and the 5-methyl group on the pyrimidine ring exhibit partial disorder. The DFT-optimized mol­ecular structure resembles the structure of the minor component in the crystal.

## Abstract 2

The crystal and mol­ecular structures of methyl 1-[(6-meth­oxy-5-methyl­pyrimidin-4-yl)meth­yl]-1*H*-benzo[*d*]imidazole-7-carboxyl­ate,obtained as a side product during the synthesis of the previously reported anti­tubercular agent *N*-(2-fluoro­eth­yl)-1-[(6-meth­oxy-5-methyl­pyrimidin-4-yl)meth­yl]-1*H*-benzo[*d*]imidazole-4-carboxamide, are reported.

## Graphical abstract

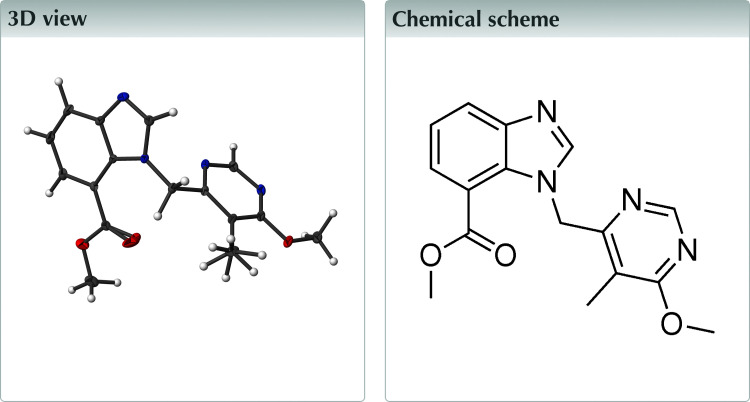

## Structure description

**Figure 1:**
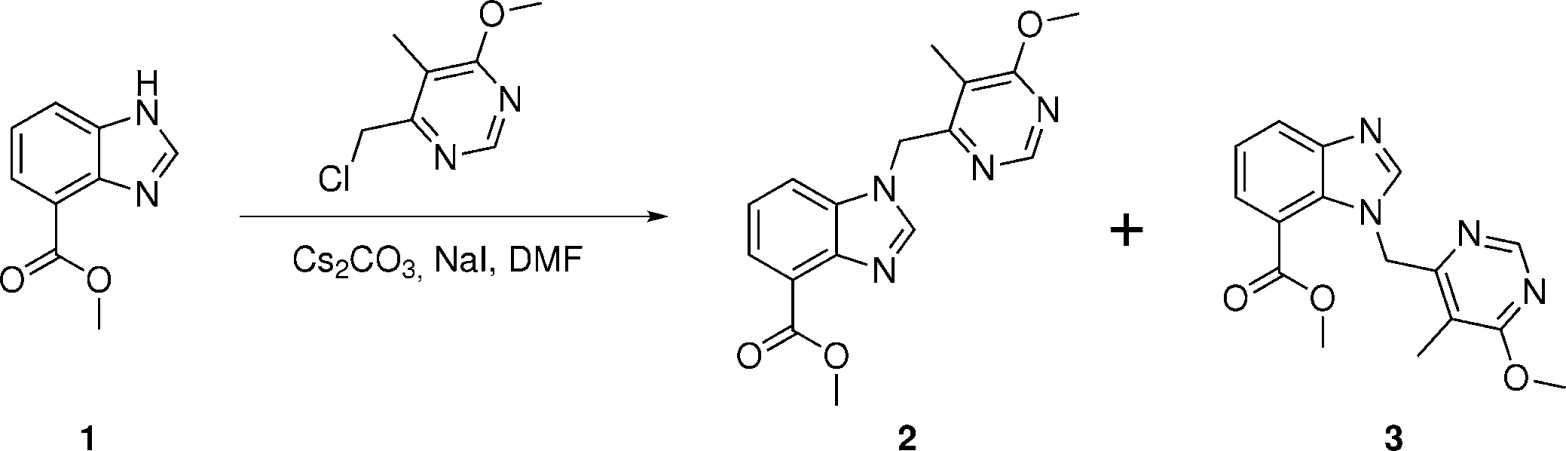
*N*-Alkyl­ation of **1** with 4-(chloro­meth­yl)-6-meth­oxy-5-methyl­pyrimidine to yield structural isomers **2** and **3**.

In the course of our studies on anti­mycobacterial agents, we synthesized and studied the compound *N*-(2-fluoro­eth­yl)-1-[(6-meth­oxy-5-methyl­pyrimidin-4-yl)meth­yl]-1*H*-benzo[*d*]imidazole-4-carboxamide (Richter *et al.*, 2022 ▸), a benzimidazole analogue of the 1,4-aza­indole-based anti­tuberculosis clinical drug candidate TBA-7371 (Shirude *et al.*, 2013 ▸, 2014 ▸), following the route published by Manjunatha *et al.* (2019 ▸). Therein, methyl 1*H*-benzo[*d*]imidazole-4-carboxyl­ate (**1**) is *N*-alkyl­ated with 4-(chloro­meth­yl)-6-meth­oxy-5-methyl­pyrimidine to yield the desired methyl 1-[(6-meth­oxy-5-methyl­pyrimidin-4-yl)meth­yl]-1*H*-benzo[*d*]imidazole-4-carboxyl­ate (**2**) and its structural isomer methyl 1-[(6-meth­oxy-5-methyl­pyrimidin-4-yl)meth­yl]-1*H*-benzo[*d*]imidazole-7-carboxyl­ate (**3**), the title compound, as a side product (Fig. 1 ▸). After separation by flash chromatography, the ratio of **2** and **3** was approximately 3.75:1 (Richter *et al.*, 2022 ▸). We have now structurally characterized compound **3** by X-ray crystallography and computational methods.

**Figure 2:**
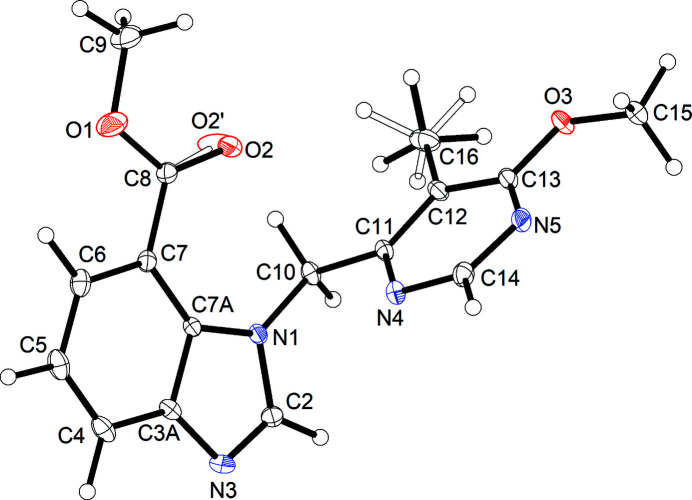
Displacement ellipsoid plot of **3** (50% probability level). Hydrogen atoms are represented by small spheres of arbitrary radius. Disordered parts with minor occupancy are drawn with empty bonds.

**Figure 3:**
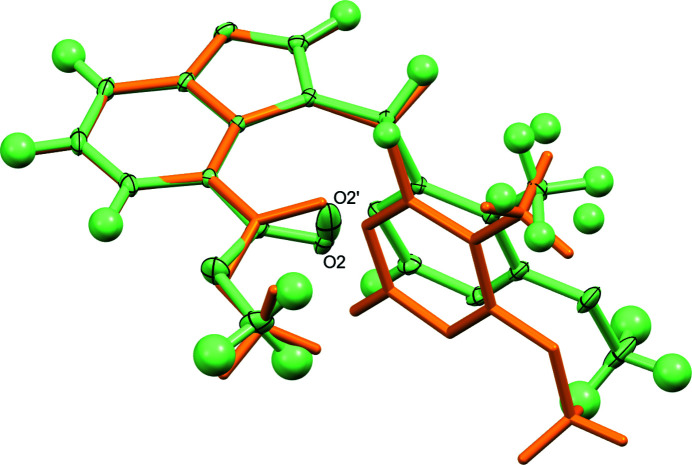
Structure overlay plot of the mol­ecular structure of **3** in the crystal (green; displacement ellipsoids with 50% probability) and the DFT-optimized mol­ecular structure (orange). The respective benzimidazole moieties were superimposed (r.m.s deviation for non-hydrogen atoms: 0.024 Å).

Fig. 2 ▸ shows the mol­ecular structure of **3** in the crystal. The mol­ecule exhibits an angular shape, similar to the aforementioned *N*-(2-fluoro­eth­yl)-1-[(6-meth­oxy-5-methyl­pyrimidin-4-yl)meth­yl]-1*H*-benzo[*d*]imidazole-4-carboxamide in the crystal (CSD refcode: DEVGEU; Richter *et al.*, 2022 ▸). In **3**, the angle between the mean planes through the benzimidazole moiety and the pyrimidine ring is 84.11 (3)°. The C2—N1—C10—C11 torsion angle is −87.61 (6)° in the chosen asymmetric unit, but the oppositely handed conformer is present in the centrosymmetric crystal structure. The methyl group on the pyrimidine ring and the carbonyl oxygen atom of the carboxyl­ate group each were found to be partially disordered over two positions. The two orientations taken up by the methyl group of C16 may cause the carboxyl­ate group to move slightly, hence the minor component O2′. To gain insight into the structural features of **3**, we optimized the structure of an isolated mol­ecule by DFT calculations. An overlay of the mol­ecular structures from X-ray crystallography and DFT structure optimization (Fig. 3 ▸) reveals that the conformation of the minor-disorder component in the crystal structure is very similar to the DFT-optimized structure. The latter exhibits a relatively short intra­molecular C—H⋯O contact between oxygen atom of the carbonyl group and one of the hydrogen atoms of the bridging methyl­ene group (O⋯H = 2.13 Å).

**Figure 4:**
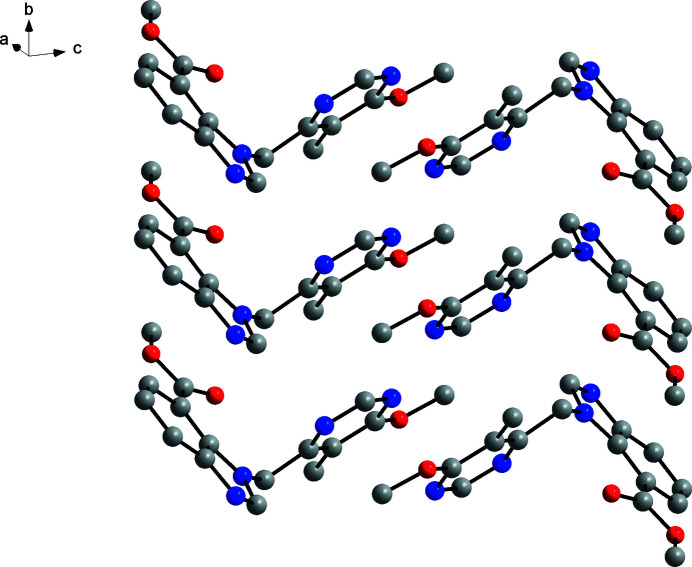
Section of the crystal structure of **3**. Colour scheme: carbon, grey; nitro­gen, blue; oxygen, red. Hydrogen atoms and O2′ are omitted for clarity.

The solid-state structure of **3** appears to be governed by close packing. The packing index calculated with *PLATON* (Spek, 2020 ▸) for the major disorder part is 73.8%, indicating a dense crystal packing (Kitaigorodskii, 1973 ▸). As shown in Fig. 4 ▸, face-to-face π–π stacking each between the benzimid­azole and pyrimidine systems of adjacent mol­ecules is a dominating structural motif. It is inter­esting to note that, in contrast to DEVGEU, the benzimidazole C2—H2 group does not form short C—H⋯*X* (*X* = N, O) contacts in the crystal structure of **3**.

## Synthesis and crystallization

We obtained compound **3** as a side product in the deliberate synthesis of its structural isomer **2**, following the route published by Manjunatha *et al.* (2019 ▸). The isomers were separated by flash chromatography (Richter *et al.*, 2022 ▸). Crystals of **3** suitable for X-ray crystallography were obtained as follows: Slow evaporation of a solution of the compound in chloro­form-*d* to dryness yielded a powder, which was redissolved in methanol. The solution thus obtained was again set aside at room temperature, and the solvent was allowed to evaporate slowly. Colourless crystals appeared after the vessel had been left undisturbed for a couple of weeks.

## Refinement

Crystal data, data collection and structure refinement details are listed in Table 1 ▸. Initial independent-atom model refinement was carried out with *SHELXL2018* (Sheldrick, 2015*b* ▸). The final structure refinement was carried out by Hirshfeld atom refinement with non-spherical atomic form factors factors using *NoSpherA2* (Kleemiss *et al.*, 2021 ▸; Midgley *et al.*, 2021 ▸) partitioning in *OLEX2* (Dolomanov *et al.*, 2009 ▸) based on electron density from iterative single-determinant SCF single-point DFT calculations using *ORCA* (version 4.1.1; Neese *et al.*, 2020 ▸) with a B3LYP functional (Becke, 1993 ▸; Lee *et al.*, 1988 ▸) and a def2-TZVPP basis set (Weigend & Ahlrichs, 2005 ▸). The carbonyl oxygen atom (O2) and the methyl hydrogen atoms on C16 were found to be disordered over two positions in each case. Standard similar distance restraints were applied to the C8—O2 and C8—O2′ distances as well as on the 1,2- and 1,3-distances of the disordered methyl hydrogen atoms. The ratio of occupancy was refined by means of a free variable for each disordered group to give 0.63 (4):0.37 (4) for the carbonyl oxygen atom and 0.646 (12):0.354 (12) for the hydrogen atoms of the methyl group. *U*_iso_ values of hydrogen atoms were refined freely, except for those affected by disorder, for which *U*_iso_(H) = 1.5*U*_eq_(C) was set.

DFT structure optimization of an isolated mol­ecule of **3** was undertaken using *ORCA* (version 5.0; Neese *et al.*, 2020 ▸) with a B3LYP(G) (VWN1) hybrid functional (20% HF exchange) (Becke, 1993 ▸; Lee *et al.*, 1988 ▸; Hertwig & Koch, 1997 ▸), using a def2-TZVPP basis set (Weigend & Ahlrichs, 2005 ▸) utilizing the auxiliary basis def2/J (Weigend, 2006 ▸). The input structure was generated from the major disorder component in the crystal structure. Optimization of the structures used the BFGS method from an initial Hessian according to Almoef’s model with a very tight self-consistent field convergence threshold (Fletcher, 2000 ▸). The optimized local minimum-energy structure exhibited only positive frequencies.

## Supplementary Material

Crystal structure: contains datablock(s) I, global. DOI: 10.1107/S2414314623000251/bt4130sup1.cif

Structure factors: contains datablock(s) I. DOI: 10.1107/S2414314623000251/bt4130Isup2.hkl

Supporting information file. DOI: 10.1107/S2414314623000251/bt4130Isup3.cdx

ATR-FT-IR spectrum of compound 3. DOI: 10.1107/S2414314623000251/bt4130sup4.pdf

Cartesian coordinates of the DFT-calculated molecular structure of compound 3. DOI: 10.1107/S2414314623000251/bt4130sup4.txt

Supporting information file. DOI: 10.1107/S2414314623000251/bt4130Isup6.cml

CCDC reference: 2235406

Additional supporting information:  crystallographic information; 3D view; checkCIF report

## Figures and Tables

**Table 1:** Experimental details

Crystal data	
Chemical formula	C_16_H_16_N_4_O_3_
*M*_r_	312.33
Crystal system, space group	Monoclinic, *P*2_1_/*n*
Temperature (K)	100
*a*, *b*, *c* (Å)	9.3758 (7), 4.7862 (3), 32.764 (2)
β (°)	96.340 (3)
*V* (Å^3^)	1461.28 (18)
*Z*	4
Radiation type	Mo *K*α
μ (mm^−1^)	0.10
Crystal size (mm)	0.19 × 0.10 × 0.02

Data collection	
Diffractometer	Bruker AXS D8 VENTURE
Absorption correction	Gaussian (*SADABS*; Krause *et al.*, 2015 ▸)
*T*_min_, *T*_max_	0.986, 0.998
No. of measured, independent and observed [*I* ≥ 2u(*I*)] reflections	125687, 6375, 5698
*R*_int_	0.047
(sin θ/λ)_max_ (Å^−1^)	0.814

Refinement	
*R*[*F* ^2^ > 2σ(*F* ^2^)], *wR*(*F* ^2^), *S*	0.039, 0.061, 1.07
No. of reflections	6375
No. of parameters	289
No. of restraints	20
H-atom treatment	H atoms treated by a mixture of independent and constrained refinement
Δρ_max_, Δρ_min_ (e Å^−3^)	0.41, −0.40

Computer programs: *APEX4* (Bruker, 2017 ▸), *SAINT* (Bruker, 2004 ▸), *SHELXT* (Sheldrick, 2015*a* ▸), *OLEX2.refine* (Bourhis *et al.*, 2015 ▸), *DIAMOND* (Brandenburg, 2018 ▸) and *Mercury* (Macrae *et al.*, 2020 ▸), *publCIF* (Westrip, 2010 ▸).

## full crystallographic data

### Crystal data

**Table:**

C_16_H_16_N_4_O_3_	*F*(000) = 656.464
*M**_r_* = 312.33	*D*_x_ = 1.420 Mg m^−^^3^
Monoclinic, *P*2_1_/*n*	Mo *K*α radiation, λ = 0.71073 Å
*a* = 9.3758 (7) Å	Cell parameters from 9856 reflections
*b* = 4.7862 (3) Å	θ = 2.2–34.0°
*c* = 32.764 (2) Å	µ = 0.10 mm^−^^1^
β = 96.340 (3)°	*T* = 100 K
*V* = 1461.28 (18) Å^3^	Needle
*Z* = 4	0.19 × 0.10 × 0.02 mm

### Data collection

**Table:**

Bruker AXS D8 VENTURE diffractometer	6375 independent reflections
Radiation source: IµS DIAMOND	5698 reflections with *I*≥ 2u(*I*)
Incoatec multilayer optics monochromator	*R*_int_ = 0.047
Detector resolution: 7.391 pixels mm^-1^	θ_max_ = 35.4°, θ_min_ = 2.2°
φ– and ω–scans	*h* = −15→15
Absorption correction: gaussian (SADABS; Krause *et al.*, 2015)	*k* = −7→7
*T*_min_ = 0.986, *T*_max_ = 0.998	*l* = −53→52
125687 measured reflections	

### Refinement

**Table:**

Refinement on *F*^2^	Primary atom site location: dual
Least-squares matrix: full	Secondary atom site location: difference Fourier map
*R*[*F*^2^ > 2σ(*F*^2^)] = 0.039	Hydrogen site location: difference Fourier map
*wR*(*F*^2^) = 0.061	H atoms treated by a mixture of independent and constrained refinement
*S* = 1.07	*w* = 1/[σ^2^(*F*_o_^2^) + (0.0015*P*)^2^ + 0.4692*P*] where *P* = (*F*_o_^2^ + 2*F*_c_^2^)/3
6375 reflections	(Δ/σ)_max_ = 0.001
289 parameters	Δρ_max_ = 0.41 e Å^−^^3^
20 restraints	Δρ_min_ = −0.40 e Å^−^^3^
8 constraints	

### Special details

**Table:**

Experimental. Crystal mounted on a MiTeGen loop using Perfluoropolyether PFO-XR75

### Fractional atomic coordinates and isotropic or equivalent isotropic displacement parameters (Å^2^)

**Table:**

	*x*	*y*	*z*	*U*_iso_*/*U*_eq_	Occ. (<1)
C2	0.41793 (7)	0.30578 (14)	0.347562 (19)	0.01418 (11)	
H2	0.3810 (9)	0.143 (2)	0.3663 (3)	0.030 (2)*	
C3A	0.43336 (6)	0.62087 (13)	0.302844 (18)	0.01290 (10)	
C4	0.40396 (7)	0.80361 (15)	0.269895 (19)	0.01730 (12)	
H4	0.2991 (10)	0.811 (2)	0.2534 (3)	0.036 (3)*	
C5	0.51499 (8)	0.96871 (15)	0.25906 (2)	0.01903 (12)	
H5	0.4962 (10)	1.114 (2)	0.2346 (3)	0.041 (3)*	
C6	0.65157 (7)	0.95258 (14)	0.280843 (19)	0.01600 (11)	
H6	0.7336 (10)	1.083 (2)	0.2723 (3)	0.038 (3)*	
C7	0.68523 (6)	0.77003 (13)	0.313947 (17)	0.01144 (10)	
C7A	0.57230 (6)	0.59893 (12)	0.324773 (17)	0.00994 (9)	
C8	0.83210 (7)	0.77743 (14)	0.336015 (19)	0.01473 (11)	
C9	1.06327 (8)	0.9716 (2)	0.33753 (3)	0.02662 (16)	
H9a	1.1186 (11)	0.774 (2)	0.3367 (3)	0.046 (3)*	
H9b	1.1135 (12)	1.129 (3)	0.3209 (3)	0.057 (3)*	
H9c	1.0648 (12)	1.029 (3)	0.3683 (4)	0.054 (3)*	
C10	0.65713 (7)	0.27497 (13)	0.385944 (18)	0.01206 (10)	
H10a	0.7645 (9)	0.270 (2)	0.3765 (3)	0.024 (2)*	
H10b	0.6245 (10)	0.064 (2)	0.3908 (3)	0.031 (2)*	
C11	0.65753 (6)	0.43198 (12)	0.426058 (17)	0.01067 (10)	
C12	0.75223 (6)	0.35346 (13)	0.459654 (18)	0.01303 (10)	
C13	0.73550 (7)	0.49763 (15)	0.496289 (18)	0.01579 (12)	
C14	0.55770 (7)	0.76028 (14)	0.464026 (19)	0.01531 (11)	
H14	0.4812 (10)	0.925 (2)	0.4657 (3)	0.034 (2)*	
C15	0.80160 (9)	0.5572 (3)	0.56782 (2)	0.0335 (2)	
H15a	0.8874 (11)	0.483 (2)	0.5890 (3)	0.048 (3)*	
H15b	0.8061 (12)	0.782 (3)	0.5644 (3)	0.053 (3)*	
H15c	0.7008 (12)	0.496 (3)	0.5774 (3)	0.053 (3)*	
C16	0.86532 (7)	0.13435 (16)	0.45806 (2)	0.02042 (13)	
H16a	0.8658 (17)	−0.003 (3)	0.4854 (4)	0.03063 (19)*	0.646 (12)
H16b	0.8514 (16)	0.001 (3)	0.4320 (4)	0.03063 (19)*	0.646 (12)
H16c	0.9698 (13)	0.230 (3)	0.4614 (5)	0.03063 (19)*	0.646 (12)
H16d	0.932 (3)	0.191 (5)	0.4335 (7)	0.03063 (19)*	0.354 (12)
H16e	0.815 (2)	−0.059 (4)	0.4467 (8)	0.03063 (19)*	0.354 (12)
H16f	0.934 (3)	0.101 (6)	0.4846 (6)	0.03063 (19)*	0.354 (12)
N1	0.55809 (5)	0.38936 (11)	0.353175 (15)	0.01077 (9)	
N3	0.33883 (6)	0.43622 (13)	0.318270 (17)	0.01624 (10)	
N4	0.56045 (6)	0.63817 (11)	0.427757 (15)	0.01239 (9)	
N5	0.63968 (6)	0.69880 (13)	0.498873 (16)	0.01738 (11)	
O1	0.91869 (5)	0.94660 (12)	0.318121 (17)	0.02457 (11)	
O2	0.8667 (4)	0.6788 (12)	0.36899 (8)	0.0202 (7)	0.63 (4)
O2'	0.8822 (11)	0.606 (5)	0.3628 (6)	0.040 (3)	0.37 (4)
O3	0.82283 (5)	0.42339 (13)	0.529660 (14)	0.02307 (11)	

### Atomic displacement parameters (Å^2^)

**Table:**

	*U*^11^	*U*^22^	*U*^33^	*U*^12^	*U*^13^	*U*^23^
C2	0.0136 (2)	0.0155 (3)	0.0133 (2)	−0.0044 (2)	0.00101 (19)	0.0002 (2)
C3A	0.0127 (2)	0.0141 (2)	0.0112 (2)	0.0007 (2)	−0.00191 (18)	−0.0002 (2)
C4	0.0193 (3)	0.0185 (3)	0.0128 (3)	0.0042 (2)	−0.0039 (2)	0.0019 (2)
C5	0.0263 (3)	0.0176 (3)	0.0127 (3)	0.0036 (3)	0.0002 (2)	0.0053 (2)
C6	0.0215 (3)	0.0139 (3)	0.0131 (2)	−0.0006 (2)	0.0042 (2)	0.0028 (2)
C7	0.0132 (2)	0.0112 (2)	0.0101 (2)	−0.0013 (2)	0.00209 (18)	−0.00018 (19)
C7A	0.0108 (2)	0.0104 (2)	0.0083 (2)	−0.00019 (19)	−0.00006 (17)	−0.00010 (18)
C8	0.0137 (2)	0.0185 (3)	0.0121 (2)	−0.0046 (2)	0.00212 (19)	−0.0016 (2)
C9	0.0180 (3)	0.0330 (4)	0.0292 (4)	−0.0117 (3)	0.0044 (3)	−0.0039 (3)
C10	0.0158 (2)	0.0102 (2)	0.0099 (2)	0.0013 (2)	−0.00020 (19)	0.00046 (19)
C11	0.0137 (2)	0.0097 (2)	0.0083 (2)	−0.00153 (19)	−0.00023 (18)	0.00081 (18)
C12	0.0137 (2)	0.0140 (3)	0.0106 (2)	−0.0028 (2)	−0.00208 (19)	0.0020 (2)
C13	0.0158 (3)	0.0217 (3)	0.0094 (2)	−0.0077 (2)	−0.00081 (19)	0.0008 (2)
C14	0.0178 (3)	0.0158 (3)	0.0128 (2)	−0.0021 (2)	0.0038 (2)	−0.0032 (2)
C15	0.0198 (3)	0.0690 (7)	0.0109 (3)	−0.0133 (4)	−0.0013 (2)	−0.0033 (4)
C16	0.0153 (3)	0.0203 (3)	0.0242 (3)	0.0007 (2)	−0.0044 (2)	0.0051 (3)
N1	0.0120 (2)	0.0113 (2)	0.00872 (19)	−0.00109 (17)	0.00008 (15)	0.00024 (17)
N3	0.0110 (2)	0.0203 (3)	0.0166 (2)	−0.0027 (2)	−0.00210 (18)	−0.0011 (2)
N4	0.0158 (2)	0.0115 (2)	0.0099 (2)	0.00026 (18)	0.00143 (17)	−0.00015 (17)
N5	0.0189 (2)	0.0234 (3)	0.0101 (2)	−0.0071 (2)	0.00264 (18)	−0.0043 (2)
O1	0.0175 (2)	0.0267 (3)	0.0297 (3)	−0.0083 (2)	0.00328 (19)	0.0061 (2)
O2	0.0140 (6)	0.0305 (12)	0.0153 (9)	−0.0053 (6)	−0.0022 (4)	0.0069 (6)
O2'	0.0159 (15)	0.054 (5)	0.047 (3)	−0.014 (2)	−0.0119 (19)	0.030 (4)
O3	0.0194 (2)	0.0376 (3)	0.01085 (19)	−0.0088 (2)	−0.00411 (16)	0.0020 (2)

### Geometric parameters (Å, º)

**Table:**

C2—H2	1.071 (9)	C10—H10b	1.074 (10)
C2—N1	1.3666 (8)	C10—C11	1.5136 (8)
C2—N3	1.3052 (8)	C10—N1	1.4472 (7)
C3A—C4	1.3931 (9)	C11—C12	1.3876 (8)
C3A—C7A	1.4204 (8)	C11—N4	1.3478 (8)
C3A—N3	1.3860 (8)	C12—C13	1.4082 (9)
C4—H4	1.069 (9)	C12—C16	1.4963 (10)
C4—C5	1.3841 (10)	C13—N5	1.3259 (9)
C5—H5	1.062 (10)	C13—O3	1.3395 (8)
C5—C6	1.3975 (10)	C14—H14	1.073 (10)
C6—H6	1.051 (10)	C14—N4	1.3271 (8)
C6—C7	1.4013 (8)	C14—N5	1.3368 (8)
C7—C7A	1.4143 (8)	C15—H15a	1.064 (11)
C7—C8	1.4834 (9)	C15—H15b	1.084 (13)
C7A—N1	1.3846 (7)	C15—H15c	1.070 (11)
C8—O1	1.3280 (8)	C15—O3	1.4381 (10)
C8—O2	1.191 (4)	C16—H16a	1.111 (11)
C8—O2'	1.255 (8)	C16—H16b	1.060 (11)
C9—H9a	1.080 (12)	C16—H16c	1.076 (11)
C9—H9b	1.069 (12)	C16—H16d	1.105 (15)
C9—H9c	1.043 (11)	C16—H16e	1.085 (15)
C9—O1	1.4374 (9)	C16—H16f	1.035 (15)
C10—H10a	1.085 (9)		
			
N1—C2—H2	120.2 (5)	C16—C12—C11	123.78 (6)
N3—C2—H2	125.1 (5)	C16—C12—C13	121.28 (6)
N3—C2—N1	114.76 (6)	N5—C13—C12	123.39 (6)
C7A—C3A—C4	122.03 (6)	O3—C13—C12	116.75 (6)
N3—C3A—C4	127.10 (6)	O3—C13—N5	119.86 (6)
N3—C3A—C7A	110.86 (5)	N4—C14—H14	116.8 (5)
H4—C4—C3A	120.3 (5)	N5—C14—H14	116.2 (5)
C5—C4—C3A	117.85 (6)	N5—C14—N4	126.99 (6)
C5—C4—H4	121.9 (5)	H15b—C15—H15a	111.3 (9)
H5—C5—C4	120.1 (5)	H15c—C15—H15a	110.2 (8)
C6—C5—C4	120.68 (6)	H15c—C15—H15b	110.5 (9)
C6—C5—H5	119.2 (5)	O3—C15—H15a	104.5 (6)
H6—C6—C5	119.1 (5)	O3—C15—H15b	109.9 (6)
C7—C6—C5	123.02 (6)	O3—C15—H15c	110.2 (6)
C7—C6—H6	117.9 (5)	H16a—C16—C12	109.1 (8)
C7A—C7—C6	116.38 (5)	H16b—C16—C12	115.1 (8)
C8—C7—C6	118.68 (6)	H16b—C16—H16a	106.4 (10)
C8—C7—C7A	124.88 (5)	H16c—C16—C12	109.8 (8)
C7—C7A—C3A	120.03 (5)	H16c—C16—H16a	104.3 (10)
N1—C7A—C3A	104.04 (5)	H16c—C16—H16b	111.6 (10)
N1—C7A—C7	135.91 (5)	H16d—C16—C12	108.1 (13)
O1—C8—C7	112.29 (6)	H16d—C16—H16a	141.4 (15)
O2—C8—C7	125.30 (16)	H16d—C16—H16b	65.9 (14)
O2—C8—O1	121.60 (15)	H16d—C16—H16c	52.4 (13)
O2'—C8—C7	125.5 (3)	H16e—C16—C12	109.2 (13)
O2'—C8—O1	120.6 (3)	H16e—C16—H16a	74.6 (14)
H9b—C9—H9a	111.2 (8)	H16e—C16—H16c	138.8 (16)
H9c—C9—H9a	107.4 (9)	H16e—C16—H16d	102.6 (15)
H9c—C9—H9b	110.5 (9)	H16f—C16—C12	117.2 (13)
O1—C9—H9a	110.4 (6)	H16f—C16—H16b	126.2 (16)
O1—C9—H9b	106.2 (6)	H16f—C16—H16c	60.3 (14)
O1—C9—H9c	111.2 (6)	H16f—C16—H16d	107.5 (15)
H10b—C10—H10a	108.2 (7)	H16f—C16—H16e	111.2 (17)
C11—C10—H10a	110.1 (5)	C7A—N1—C2	106.50 (5)
C11—C10—H10b	108.1 (5)	C10—N1—C2	121.18 (5)
N1—C10—H10a	109.6 (5)	C10—N1—C7A	132.16 (5)
N1—C10—H10b	107.3 (5)	C3A—N3—C2	103.82 (5)
N1—C10—C11	113.34 (5)	C14—N4—C11	116.07 (5)
C12—C11—C10	119.63 (5)	C14—N5—C13	115.79 (5)
N4—C11—C10	117.55 (5)	C9—O1—C8	116.61 (6)
N4—C11—C12	122.78 (5)	C15—O3—C13	117.44 (7)
C13—C12—C11	114.94 (6)		
			
C2—N1—C7A—C3A	−1.20 (5)	C6—C7—C8—O2	163.8 (3)
C2—N1—C7A—C7	−179.47 (5)	C6—C7—C8—O2'	−171.1 (18)
C2—N1—C10—C11	−87.61 (6)	C7—C7A—N1—C10	5.16 (9)
C2—N3—C3A—C4	177.48 (5)	C7—C8—O1—C9	179.13 (6)
C2—N3—C3A—C7A	−1.12 (6)	C7A—N1—C10—C11	87.20 (7)
C3A—C4—C5—C6	−0.44 (8)	C10—C11—C12—C13	−174.93 (6)
C3A—C7A—C7—C6	−0.92 (7)	C10—C11—C12—C16	5.43 (7)
C3A—C7A—C7—C8	176.33 (5)	C10—C11—N4—C14	176.03 (5)
C3A—C7A—N1—C10	−176.57 (4)	C11—C12—C13—N5	−1.68 (7)
C4—C5—C6—C7	0.90 (8)	C11—C12—C13—O3	178.54 (5)
C5—C6—C7—C7A	−0.18 (8)	C11—N4—C14—N5	−0.65 (7)
C5—C6—C7—C8	−177.62 (6)	C12—C13—N5—C14	−0.16 (7)
C6—C7—C7A—N1	177.14 (5)	C12—C13—O3—C15	−176.49 (6)
C6—C7—C8—O1	−5.89 (6)	C13—N5—C14—N4	1.46 (7)
