# Supplementary figures and images for: Methyl 1-[(6-meth­oxy-5-methyl­pyrimidin-4-yl)meth­yl]-1H-benzo[d]imidazole-7-carboxyl­ate: a combined X-ray and DFT study
Source: IUCrdata. 2023 Jan 12;8(Pt 1):x230025. doi: 10.1107/S2414314623000251 (PMC9912325; doi:10.1107/S2414314623000251)

## GOD-GB-148

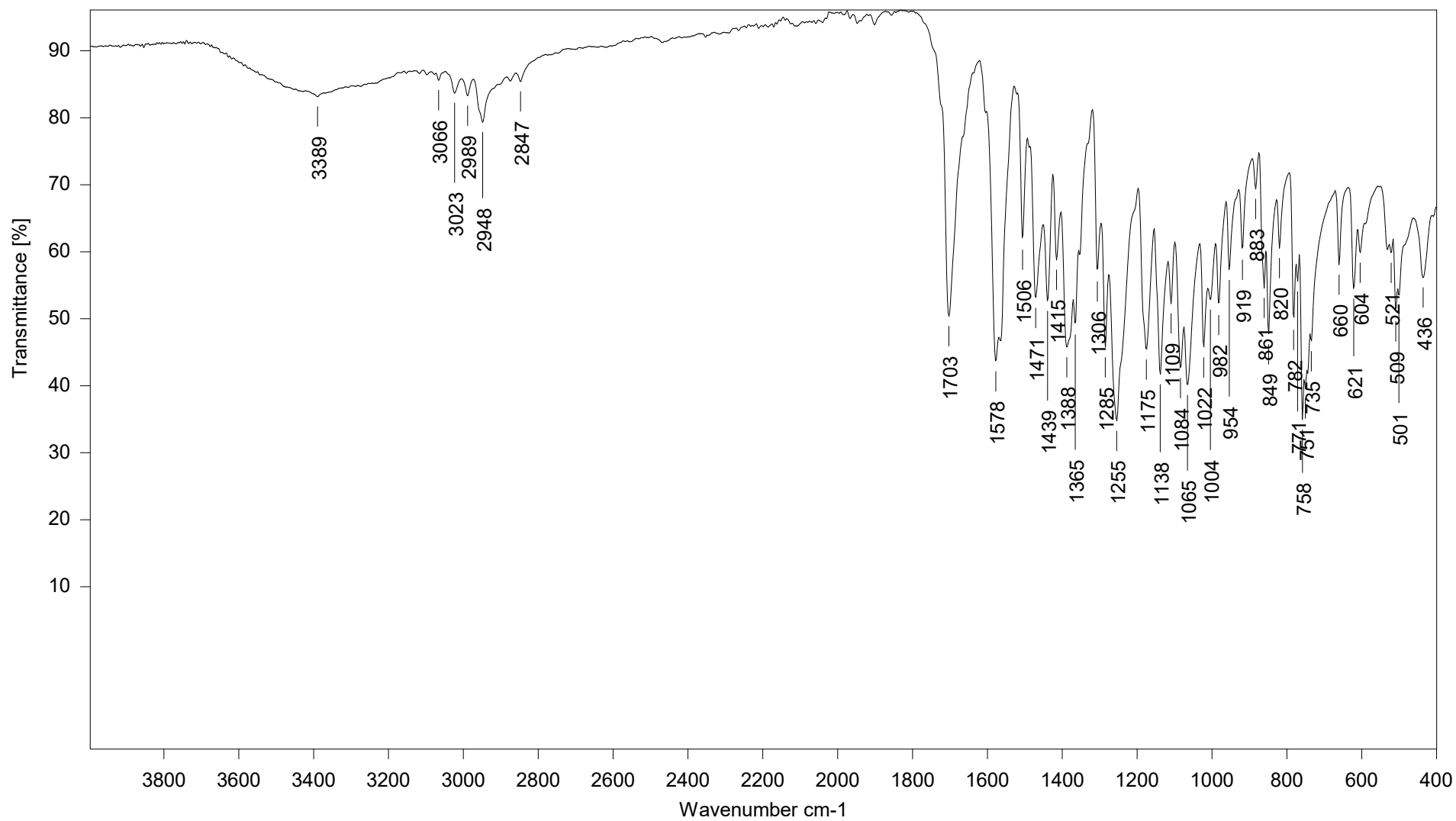

Supplement: Supplementary file 4 [file x-08-x230025-sup4.pdf]
